# Deoxycalyciphylline B, a Hepatotoxic Alkaloid from *Daphniphyllum calycinum*

**DOI:** 10.3390/molecules17089641

**Published:** 2012-08-13

**Authors:** Xiaopo Zhang, Junqing Zhang, Yinfeng Tan, Qibing Liu, Mingsheng Liu

**Affiliations:** 1 School of Pharmaceutical Science, Hainan Medical University, Haikou 571101, Hainan, China; Email: xiaopozhang@yahoo.com (X.Z.); jqzhang2011@163.com (J.Z.); secondmessenger@163.com (Y.T.); qibingliu@yahoo.com.cn (Q.L.); 2 Hainan Provincial Institute of South and Li Medicine Development, Haikou 571101, Hainan, China; 3 Guangzhou Unversity of Chinese Medicine, Guangzhou 510006, Guangdong, China

**Keywords:** *Daphniphyllum calycinum*, hepatic toxicity, alkaloid, deoxycalyciphylline B

## Abstract

*Daphniphyllum calycinum* (DC), a main component of Chinese patent drug Fengliao-Changweikang, is effectively used to cure bowel disease in the clinic. It was recorded that DC possessed slight toxicity, which was caused by the alkaloids existing in its extract. Unfortunately, to date the toxicity level and toxic constituents are still unclear. The present study is designed to illustrate the acute toxicity and induced organ damages of the total alkaloids as well as to determine the toxic constituents. Based on the above studies, not only was the acute toxicity determined but also hepatic toxicity was characterized by increased plasma biomarkers of ALT and AST and liver cell inflammatory infiltrate as well necrosis that was firstly observed. Significantly, deoxycalyciphylline B exhibited exactly the same hepatic toxicity so it was identified as the main toxic constituent in DC. An obvious dose-effect relationship between the toxic compound and induced hepatic injuries was also observed. Moreover, the Chinese patent drug Fengliao-Changweikang contained low levels of the toxic compound, compared with the total alkaloids. Therefore, this Chinese patent drug could be regarded to be safe in this point of view.

## 1. Introduction

*Daphniphyllum* alkaloids are characterized by highly complex frameworks with unique polycyclic skeletons that have attracted great interest in their isolation and total synthesis as well as biosynthetic studies. *Daphniphyllum calycinum *Benth., which is rich in *Daphniphyllum* alkaloids, is also a traditional Chinese medicine used to cure many diseases. Moreover, the extract of this herbal medicine is an important component of the Chinese patent drug Fengliao-Changweikang, which is effective in curing enterogastritis [1]. Previous records and studies indicated that this plant is slightly poisonous, and the toxicity was induced by the alkaloids present [2,3]. This was further confirmed in our previous studies. In our former study, both the water extract of DC and Fengliao Changweikang showed no actue toxicity at doses of more than 200 times the daily dose by human and no organic damages were observed, while the 95% ethanol extract of DC (about 50 times of normal daily dose) showed slight toxicity with a few deaths and slight hepatic injuries being observed in mice. However, the toxicity level and toxic constituents are still unclear to date. This paper is designed to determine the acute toxicity level and induced organ injuries by the alkaloid in mice. The obtained alkaloids were also throughly screened *in vivo *by histology analysis of the mices’ livers in order to figure out the toxic constituents.

## 2. Results and Discussion

### 2.1. Chemical Analysis of the Total Alkaloid

**Figure 1:**
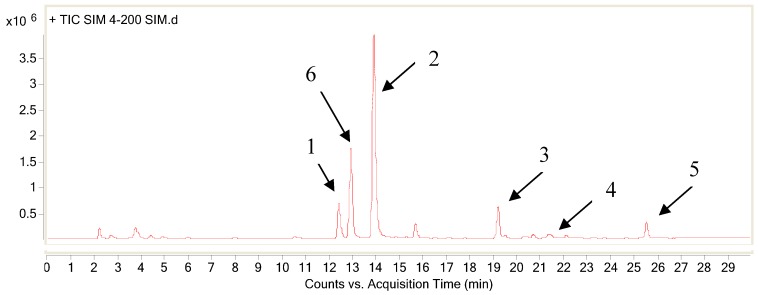
Total ion chromatogram of the total alkaloid; the arrows point to the peaks corresponding to the structures listed in Figure 2.

Phytochemical analysis of the total alkaloid was performed by HPLC-MS^n^ and five main alkaloids (compounds **1**, **2**, **3**, **4** and **5**) were assigned by comparing their MS spectral data with related literatures [4,5,6,7,8]. The total ion chromatogram was shown in Figure 1 and the MS^n^ spectra of the alkaloids are depicted in the supplemental material (Figures S1–S5).

### 2.2. Identification of the Alkaloids

**Figure 2:**
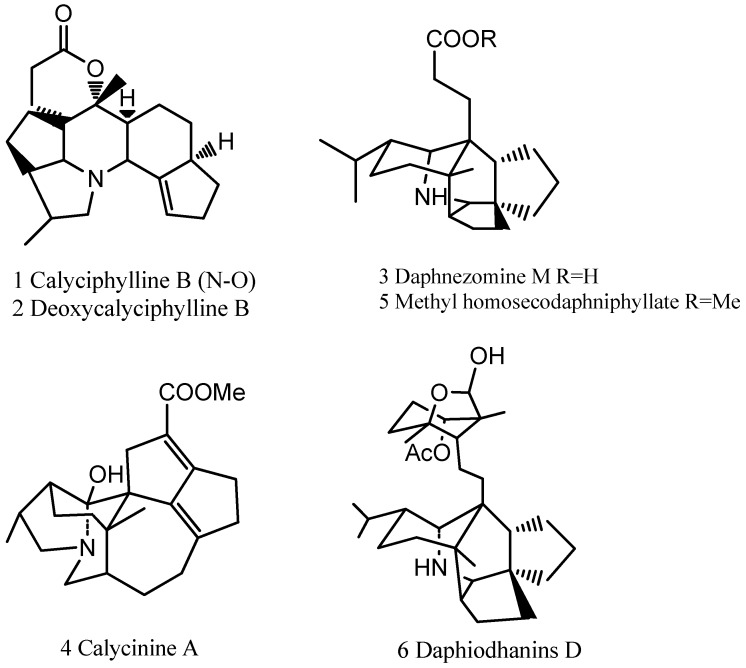
Structures of the six alkaloids determined in the total alkaloid.

Compounds **1**, **2**, **3**, **5** and **6** were isolated and purified from the total alkaloid and identified on basis of their NMR and MS data according to related literature [4,5,6,7,8,9]. Their spectral data were listed in the supplemental material and the structuress of compounds **1**–**6** are depicted in Figure 2.

### 2.3. Acute Toxicity

#### 2.3.1. Behavioral Observations

Mice treated with the total alkaloid showed behavioral changes such as gait disturbance, muscle shakes, creeping, slow response to external stimuli and rapid breathing at some tested doses starting from 15 min after administration and continuing for 30 min after they became visible. After that, everything was restored to normal in survivors. Compounds **1**, **2**, **3**, **6** were given to mice orally under the low dose of 160 mg/kg and high dose of 640 mg/kg, respectively (doses were determined by preliminary tests). Mice treated with compound **2** displayed creeping and slow response at the high dose. However, no deaths occurred at all the tested doses and the behavioral changes were observed starting from 15 min after administration. After that, everything was restored to normal levels in the animals.

#### 2.3.2. The Acute Toxicity of Total Alkaloid

In our former study, both the water extract of DC and Fengliao Changweikang showed no actue toxicity at a dose of more than 200 times the daily dose in humans and no organic damages were observed in mice, while the 95% ethanol extract of DC (about 50 times of normal daily dose) showed slight toxicity with a few deaths and slight hepatic injuries being observed in mice. The toxic extract was then purified to obtain the total alkaloid. Furthermore, by checking the acute toxicity data of the total alkaloid in mice, all of the mice died under a dose of 2,000 mg/kg, and no mice died under a dose of 300 mg/kg. The LD_50_ dose of was calculated to be 812 mg/kg with 764–860 mg/kg as the 95 percent confidence interval using a modified Karber method [10].

### 2.4. Histological Analysis

#### 2.4.1. Histological Analysis Induced by Total Alkaloid

**Figure 3:**
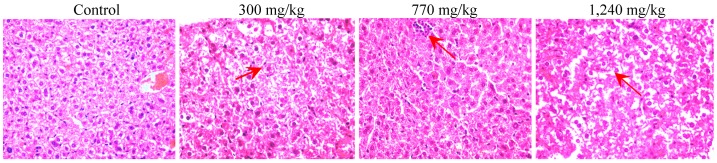
Representative histopathological changes of haematoxylin and eosin (H&E) stained liver sections of mice (original magnification 40×) from mice receiving total alkaloid; Red arrows indicated the pathologic change of inflammatory infiltrate and cell necrosis.

Results of pathological sections suggested that toxic damages occurred in the liver, and no injures appeared in the kidney and cerebellum of the mice. The hepatic injuries showed an obvious dose-effect relationship. Detailed observations were that slight inflammatory infiltrate displayed in the livers treated with 300.0 mg/kg total alkaloid, and the intercellular space became wider as well as focal cell necrosis occurred under a dose of 770.0 mg/kg. Furthermore, severe inflammatory infiltration and cell necrosis were found in groups treated with 1,240.0 mg/kg of total alkaloid, as shown in Figure 3.

#### 2.4.2. Hepatic Injuries Induced by Alkaloids

**Figure 4:**
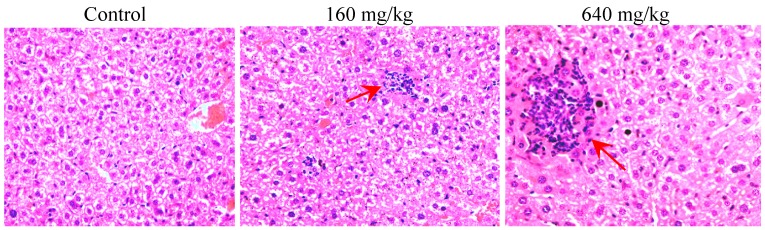
Representative histopathological changes of haematoxylin and eosin (H&E) stained liver sections of mice (original magnification 40×) treated with deoxycalyciphylline B; Red arrows showed hepatic injuries of cell necrosis and clusters of foamy cells.

Eight test groups received doses of 160 and 640 mg/kg body weight (the dose was determined by preliminary tests) of compounds **1**, **2**, **3** and **6**, respectively. Pathological check suggested that only injuries occurred in the liver of the test group treated with deoxycalyciphylline B, and no hepatic injuries appeared by oral consumption of the other alkaloids. Furthermore, the induced hepatic damages showed an obvious dose-effect relationship. Detailed pathologic changes could be summarized as slight edema occurred in the mice livers treated with 160 mg/kg deoxycalyciphylline B, more severe cell necrosis and clusters of foamy cells were found under the dose of 640 mg/kg as shown in Figure 4. Therefore, deoxycalyciphylline B was undoubtedly assigned as the main toxic constituent in the total alkaloid of DC.

**Figure 5:**
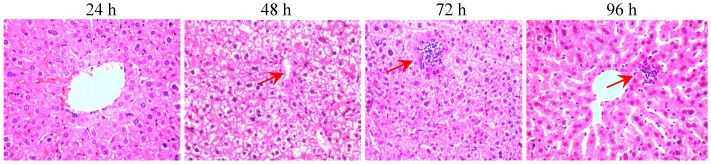
Representative histopathological changes of haematoxylin and eosin (H&E) stained liver sections of mice (original magnification 40×) treated with deoxycalyciphylline B (320 mg/kg) for 1, 4, 8, 12, 24, 48, 72 and 96 h, respectively. Red arrows showed hepatic injuries of cell necrosis or clusters of foamy cells.

Mice were divided into control group and model groups. Model groups were divided into four different time groups (n = 6). Pathological checks were carried out at different times after receiving the high dose of dexycalyciphylline B (320 mg/kg) as shown in Figure 5. The liver pathologic examination showed that slight edema occurred 24 h after the administration of the toxic compound in mice. Furthermore, serious damages were found by 96 h after the treatment, whereby severe cell necrosis and clusters of foamy cells appeared.

### 2.5. Observation of Serum Levels of AST and ALT

Plasma biochemistry measurements of aspartate aminotransferase (AST), alanine aminotransferase (ALT) were conducted. In parallel with the histological observation, those toxic events were also confirmed by the alteration of liver function markers. 1 h after the administration of deoxycalyciphylline B, the tested levels of AST and ALT in the serum of mice increased significantly (*p* < 0.01). The highest concentration was found 8 h after adminstration and the hepatoxicity lasted for more than 48 h as depicted in Table 1. 

**Table 1:** Serum levels of ALT and AST following adminstration of deoxycalyciphylline B (320 mg) and total alkaloid (600 mg) at different times in mice (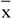 ± s, n = 10).

	ALT		AST	
	Deoxycalyciphylline B	Total alkaloid		Total alkaloid
Control	29.0 ± 5.2	29.0 ± 5.2		125.8 ± 18.2
1 h	86.4 ± 14.2 **	96.2 ± 13.6 **	164.5 ± 22.4 **	178.2 ± 25.7 **
2 h	154.8 ± 38.4 **	136.8 ± 26.4 **	312.2 ± 30.2 **	304.0 ± 32.6 **
4 h	260.6 ± 42.6 **	192.6 ± 40.2 **	393.2 ± 42.1 **	352.6 ± 38.4 **
8 h	320.8 ± 55.1 ***	230.8 ± 48.4 **	485.6 ± 60.2 ***	402.7 ± 56.8 ***
12 h	300.6 ± 50.6 ***	210.6 ± 35.6 **	460.2 ± 58.4 ***	398.2 ± 48.2 ***
24 h	222.7 ± 45.2 **	140.7 ± 25.8 **	380.2 ± 41.5 ***	310.4 ± 38.5 **
48 h	128.6 ± 32.4 **	88.6 ± 15.4 **	286.4 ± 36.4 **	250.1 ± 34.4 **
72 h	84.3 ± 13.2 **	56.3 ± 10.6 **	168.2 ± 24.2 **	188.6 ± 28.6 **

The asterisks indicate significance of differences (** *p* < 0.01, *** *p* < 0.001) in comparison to control.

**Figure 6:**
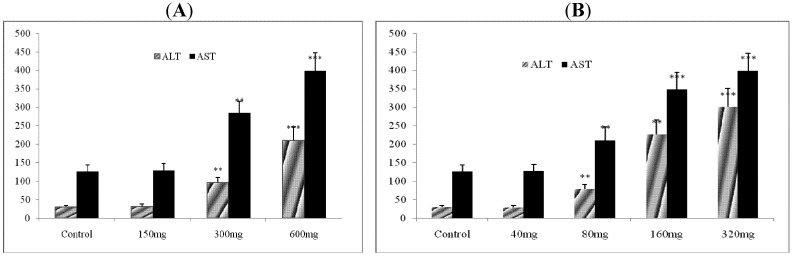
Levels of serum biomarkers of hepatotoxicity. (**A**) ALT and AST levels were estimated in serum following the administration of total alkaloid at different doses; (**B**) ALT and AST levels were estimated in serum following the administration of deoxycalyciphylline B at different doses. The asterisks indicate significance of differences (** *p* < 0.01; *** *p* < 0.001) in comparison to control.

Since the highest concentrations of serum biomarkers were found at 12 h after the treament, animals were treated with total alkaloid and deoxycalyciphyline B at different doses (n = 10) and sacrificed 12 h after the treatment. Serum ALT and AST levels were estimated following the admininstration of different doses of total alkaloid and deoxycalyciphylline B. Significant increase of serum ALT and AST were observed in 150 mg/kg dose group of total alkaloid and 80 mg/kg dose gourp deoxycalyciphylline B as compared to control (** *p* < 0.01). An obvious dose-effect of both the extract and compound were also demonstrated in Figure 6.

### 2.6. Alkaloids in Samples Treated with Different Extraction Process

**Figure 7:**
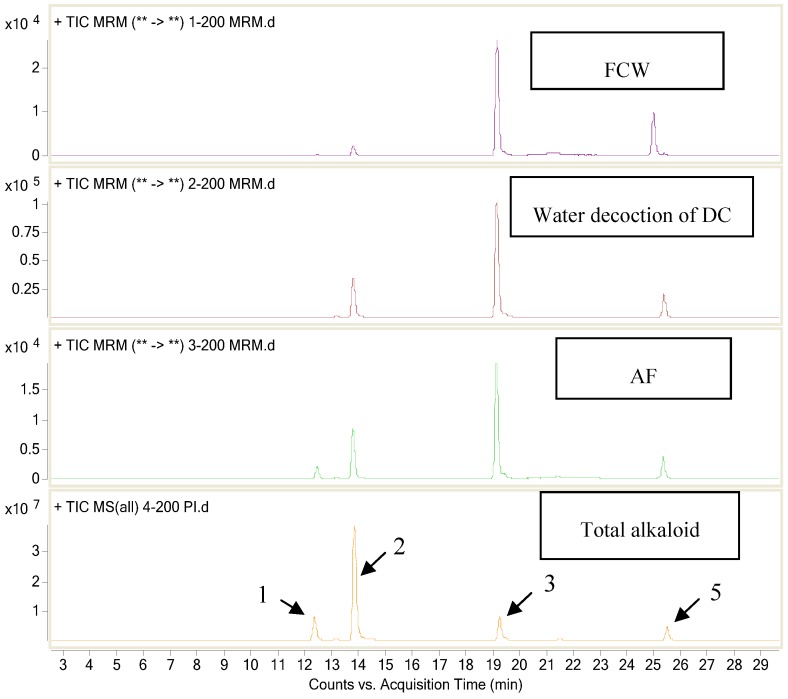
MRM chromatograms of samples treated with different extraction process; FCW equal to Chinese patent drug Fengliao Changweikang; AF equals to anti-inflammatory fraction obtained from water decoction of DC; hepatoxic component deoxycalyciphylline B showed a relative low level in FCW, water decoction of DC, and the AF.

Four samples of total alkaloid, water decoction of DC, anti-inflammatory active fraction obtained from water decoction of DC (AF), and the drug Fengliao-ChangweiKang (FCW) were dissolved in MeOH (concentration equals to the same amount of the crude material) and injected into LC-MS, separately. The multiple reaction monitoring (MRM) chromatograms of the Fengliao-ChangweiKang, water decoction of DC, and AF were compared with total alkaloid. The results suggested that deoxycalyciphylline B exhibited a relative lower level in the three samples of FCW, water decoction of DC and AF than in total alkaloid. In contrast, the content of compounds **3** and **5** exhibited a relative high level as shown in Figure 7. The detected relative low content level of deoxycalyciphylline B in Fengliao-ChangweiKang demonstrated that the drug is safety for patient administration from this point of view.

### 2.7. The Content of Deoxycalyciphylline B in Different Samples

The levels of deoxycalyciphylline B in different samples of total alkaloid, water decoction of DC, and Fengliao Changweikang was determined by applying a HPLC-MS method. The results are listed in Table 2.

**Table 2:** Content of deoxycalyciphylline B in different samples (%).

Total alkaloid	95% Ethanol extract	Water decotion of DC	Fengliao Changweikang
17.24	0.72	0.08	0.01

## 3. Experimental

### 3.1. Animals

In all the experiments, six week old male and female Kunming (KM) mice with weight of 20–22 g were kept at a constant temperature (25 °C) with a 12 h light-dark cycle with free access to water and the assigned diets for the length of each experiment. Mice were randomly allocated the following groups containing ten animals of each dose:

Group I: Vehicle (1% DMSO in distilled water) controls;
Group II: Different doses of total alkaloid (300, 770, 1,240, 2,000 mg/kg);
Group III: Different doses of obtained alkaloids (160 and 640 mg/kg);
Group IV: Different doses of total alkaloid (150, 300, 600 mg/kg);
Group V: Different doses of deoxycalyciphylline B (40, 80, 160 and 320 mg/kg).

### 3.2. Chemicals and Reagents

HPLC-grade methanol was purchased from Fisher Scientific UK Ltd (Loughborough, UK) and water was deinoized by filtering through a Hitech-Sciencetool Master-S system (Shanghai, China). Analytical grade reagents of chloroform, ethyl acetate, methanol, sulfuric acid, sodium carbonate were purchased from Beijing Chemical Works (Beijing, China). Al_2_O_3_ (100–200 mesh) was purchased from Qingdao Haiyang Chemical Co., Ltd (Shandong, China). Hematoxylin and eosin were purchased from Ricca Chemical Company (Arlington, TX, USA). Sephadex LH-20 was bought from Pharmacia (Uppsala, Sweden). The NMR data were recorded on an apparatus of Bruker AV 300 and the MS spectra were measured on LTQ Orbitrap XL spectrometer. AST and ALT were purchased from Shino-test Corporation (Sagamihara, Japan).

### 3.3. Plant Material

The bark and leaves of *Daphniphyllum calycinum *were collected in Haikou in July 2010, and the identity of the sample was confirmed by our plant taxonomy research group. A voucher specimen (No. DC-20100712) was deposited at the School of Pharmaceutical Science, Hainan Medical University. 

### 3.4. Preparation of the Total Alkaloid, Water Decoction of DC and AF

The air-dried bark and leaves of DC was exhaustively extracted with 95% ethanol (300 L, 3 h, 90 °C) after grinding into powder (28 kg). The solvent was removed under reduced pressure to afford an extract (3.2 Kg). Then the extract was suspended in water, and the pH of the suspension was adjusted to 3 by adding a solution of 2 N H_2_SO_4_. The acidic solution was next partitioned with ethyl acetate to remove the liposoluble impurities. After finishing this process, the pH of the mother liquor was adjusted to 10 by adding a solution of 2 N Na_2_CO_3_. This basic solution was partitioned with chloroform, and the extract was concentrated under reduced pressure to give the total alkaloid (28.4 g).

The air-dried stem and leaves of DC (10 kg) was extracted with water (200 L, 2 h, 98 °C) after grinding into powder. The solvent was removed under reduced pressure to afford the water decoction of DC (800 g). Then a part of the detection was subjected to HP-20 column chromatography using H_2_O and mixture solvent (H_2_O/EtOH = 3:7) successively as the eluents. The obtained H_2_O elution was discarded. The mixture solvent eluent was collected and concentrated to an extract under reduced pressure, which has been previously assigned as the active fraction of the DC (AF, 50 g). 

### 3.5. Phytochemical Analysis of the Total Alkaloid

The chemical components of the total alkaloid were analyzed on an Agilent 1200SL RRLC-6410 QQQ spectrometer (Agilent Technologies, Palo Alto, CA, USA) was connected to an Agilent 1100 HPLC instrument via an ESI ionization source in a post-column splitting ratio of 2:1. The sample dissolved in methanol, and solution was injected into the apparatus. The column used in the experiment is Agilent RRHT ZORBAX Eclipse (2.1 × 100 mm, 1.8 µm), the flow rate was 0.2 mL/min, the column temperature is 30 °C, and the mobile phase is the mixture of methanol (20) and 1% formic acid (80). The parameters of the ionization source are as follows: the analysis was performed under positive ion model, the gas temperature is 350 °C, the gas flow rate is 9 L/min, the pressure of nebulizer is 30 psi, and the fragmentor was set at 135 v.

### 3.6. Isolation and Identification of the Alkaloids

The total alkaloid was separated by column chromatography on neutral alumina using different proportions of *n*-hexane-acetone-ethanediamine as the eluent. Five fractions (Fractions 1–5) were obtained. Fraction 1 was further purified using a silica gel column chromatography to obtain compound **6** (1,000 mg). Fraction 2 was further separated using a column of Sephadex LH-20 to give compound **5** (50 mg). Compounds **2** (600 mg) and **3** (260 mg) were obtained by silica gel column and Sephadex LH-20 chromatography, respectively. Compound **1** (250 mg) was obtained from Fraction 4 by silica gel column chromatography. The structures of the isolated compounds were determined by comparing their NMR and MS data with related literatures.

### 3.7. Determination of the Content of Deoxycalyciphylline B in Different Sample

The content of deoxycalyciphylline B in different samples of total alkaloid, 95% ethanol extract, water decoction of DC, and Fengliao Changweikang was determined on a Agilent 1200SL RRLC-6410 QQQ spectrometer using the methods established above.

### 3.8. Acute Toxicity of the Total Alkaloid in Mice

In order to study the possible toxic effects and changes in normal behavior, 60 mice were divided into six groups (five males and five females, weight 20.0–22.0 g) and each was used in this experiment. The control group received the vehicle (1% DMSO in distilled water), and test groups received arithmetic doses of 300, 480, 770, 1,240, and 2,000 mg/kg body weight of the total alkaloid. Those doses were chosen after several preliminary screenings on mice. The experimental animals were deprived of food for 18 h prior to the administration of total alkaloid. They were monitored continuously for 2 h thereafter for any signs of toxicity such as reduction in locomotion, aggressiveness, reaction to stimuli (tail pinch, noise), social interactions, aspect of feces and mortality. After this period, the animals were supplied food and water *ad libitum*. Dead animals in each group were counted within 48 h following the administration of the extract and the LD_50_ was calculated by using the modified Karber method.

### 3.9. Liver Histology

Surviving mice were observed and executed after administration of the total alkaloid and the obtained alkaloids at different times. Organs of liver, kidney, and cerebellum were quickly removed and then washed by physiological saline. After that, they were fixed immediately in 4% neutral formalin and then, embedded in paraffin. Tissue sections of 5–6 mm were made from representative region of the organs by the conventional tissue preparation methods, and viewed under a light microscope (Olympus CH02, Tokyo, Japan) after hematoxylin and eosin (H&E) staining. Any alterations compared to the normal structure were registered.

### 3.10. Observation of Serum Levels of AST and ALT

Blood was taken from the mouse eye socket with heparin as an anticoagulant. The plasma obtained from each mouse was frozen at −20 °C until measurement (within 12 h). Serum was separated and AST and ALT were estimated using an automated biochemical analyzer (Beckman Coulter, CA, USA).

### 3.11. Statistical Analysis

The values were expressed as mean ± standard deviation. For each parameter, the One-Way ANOVA was used to detect the differences between the groups. When the significant differences existed, the Waller-Duncan test (*p* < 0.01) was used to compare the means.

## 4. Conclusions

In the present study, the main toxic constituent deoxycalyciphylline B was unambiguously assigned for the first time, which was mainly responsible for the hepatoxicity of DC. Furthermore, the content of the toxic constituent in samples treated with different extraction methods were also compared. Interestingly, the Chinese patent drug Fengliao Changweikang was found to have a relatively low level of deoxycalyciphylline B, which ensures its safety for patients from this point of view. Additionally, the toxicity mechanism of deoxycalyciphylline B will be investigated in the near future.

## Supplementary Materials

Supplementary materials can be accessed at: http://www.mdpi.com/1420-3049/17/8/9641/s1.

## References

[1] Liu M.S., Liu C., Zhang X.P., Sheng L., Zhang J.Q., Kang S.L. (2010). Two new drimane sesquiterpenoids from compound Changweikang and their inhibitory activity against nitric oxide production. Chem. Pharm. Bull..

[2] Liu M.S. (2008). Introduction of Li Nationality Medicine.

[3] Fang D.S., Zhou W., Chen Y., Zhu R.H. (1964). New alkaloids from *Daphniphyllum calycinum*. Acta Chin. Sin..

[4] Morita H., Yoshida N., Kobayashi J. (1999). Daphnezomines C, D and E, New alkaloids with an N-oxide moiety from *Daphniphyllum humile*. Tetrahedron.

[5] Morita H., Yoshida N., Kobayashi J. (2002). Daphnicyclidins J and K, Unique polycyclic alkaloids from Daphniphyllum humile. J. Org. Chem..

[6] Morita H., Takatsu H., Kobayashi J. (2003). Daphnezomines P, Q, R and S, New Alkaloids from *Daphniphyllum humile*. Tetrahedron.

[7] Yang S.P., Yue J.M. (2003). Two novel alkaloids with a unique fused hexacyclic skeleton from *Daphniphyllum subverticillatum*. J. Org. Chem..

[8] Zhang H., Yang S.P., Fan C.Q., Ding J., Yue J.M. (2006). Daphniyunnines A-E, Alkaloids from *Daphniphyllum yunnanense*. J. Nat. Prod..

[9] Mu S.Z., Yang X.W., Di Y.T., He H.P., Wang Y., Wang Y.H., Li L., Hao X.J. (2007). Secophnane-type alkaloids from *Daphniphyllum oldhami*. Chem. Biodivers..

[10] Chen Q. (2006). Pharmacological Research Methods of Chinese Matetia Medica.

