# Supplementary material for: Deoxycalyciphylline B, a Hepatotoxic Alkaloid from Daphniphyllum calycinum
Source: Molecules. 2012 Aug 13;17(8):9641–51. doi: 10.3390/molecules17089641 (PMC6268957; doi:10.3390/molecules17089641)
Supplement: Supplementary file 1 [file molecules-17-09641-s001.pdf]

Supplementary Material

Figure 1S. MS^3 spectrum of $[M+H]^+$ ion at *m/z* 358 of compound **1**.

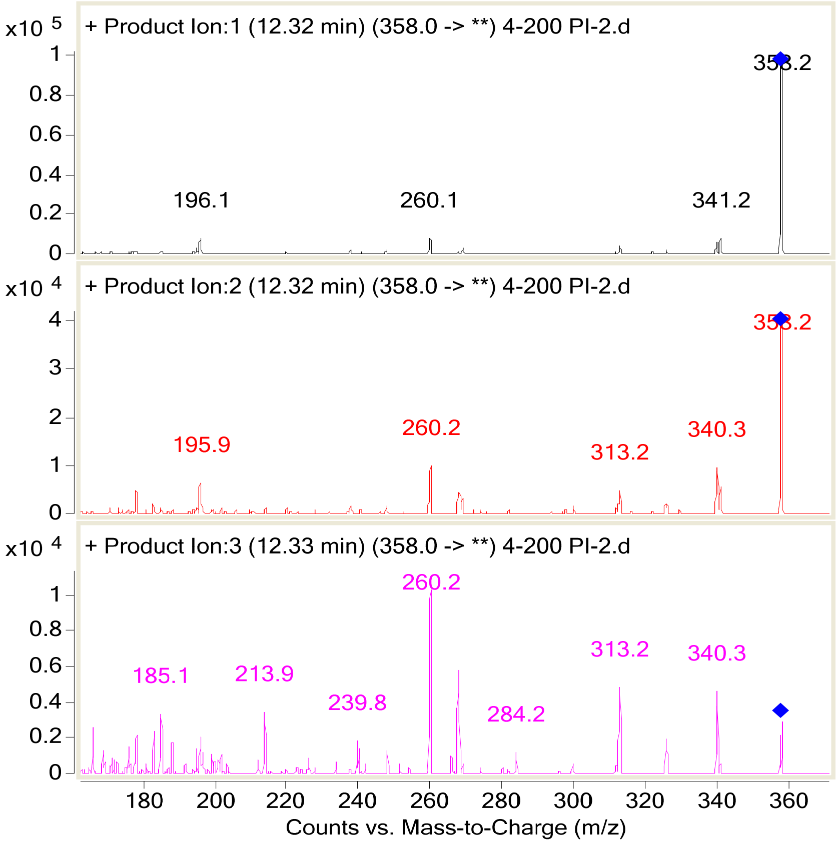

Figure 2S. MS^3 spectrum of $[M+H]^+$ ion at *m/z* 342 of compound **2**.

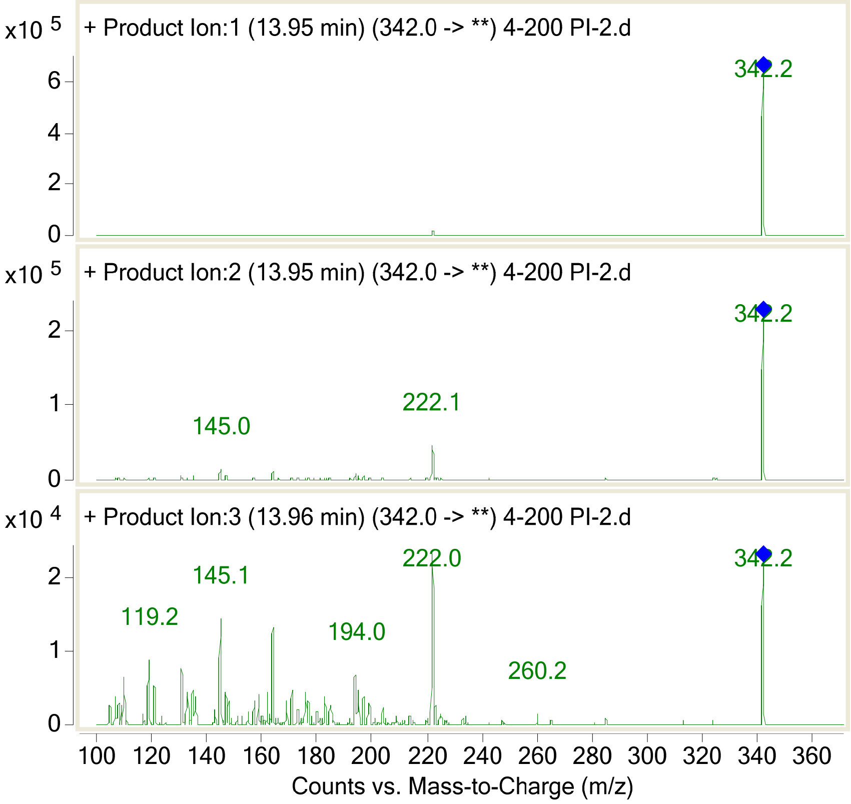

Figure 3S. MS^3 spectrum of $[M+H]^+$ ion at *m/z* 346 of compound **3**.

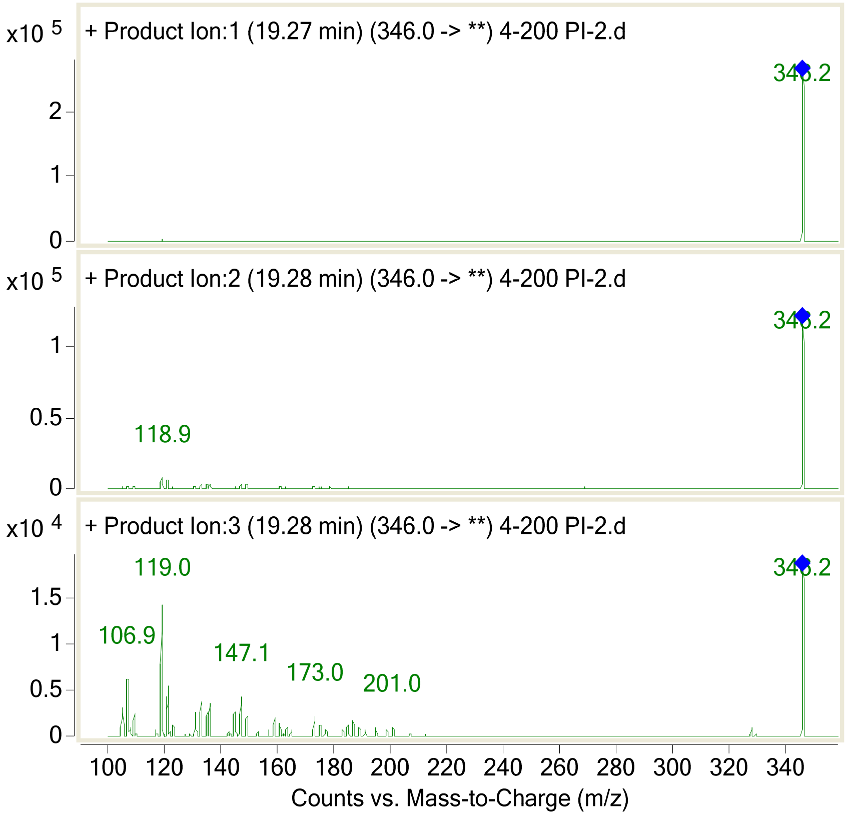

Figure 4S. MS^3 spectrum of $[M]^+$ ion at *m/z* 369 of compound **4**.

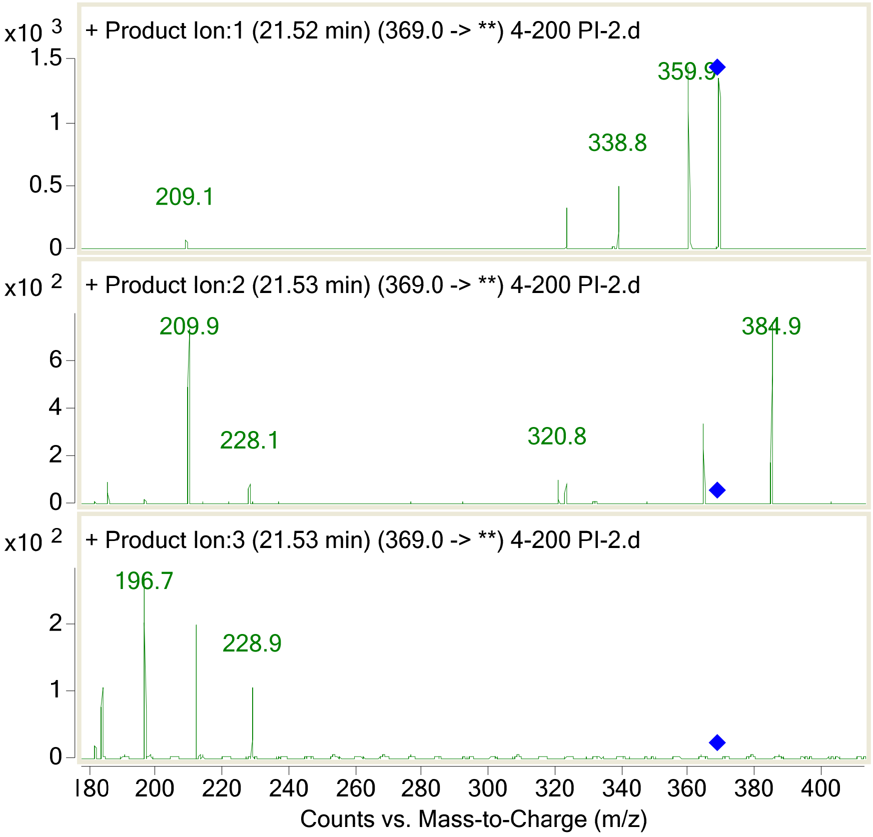

Figure 5S. MS^3 spectrum of $[M+H]^+$ ion at *m/z* 360 of compound **5**.

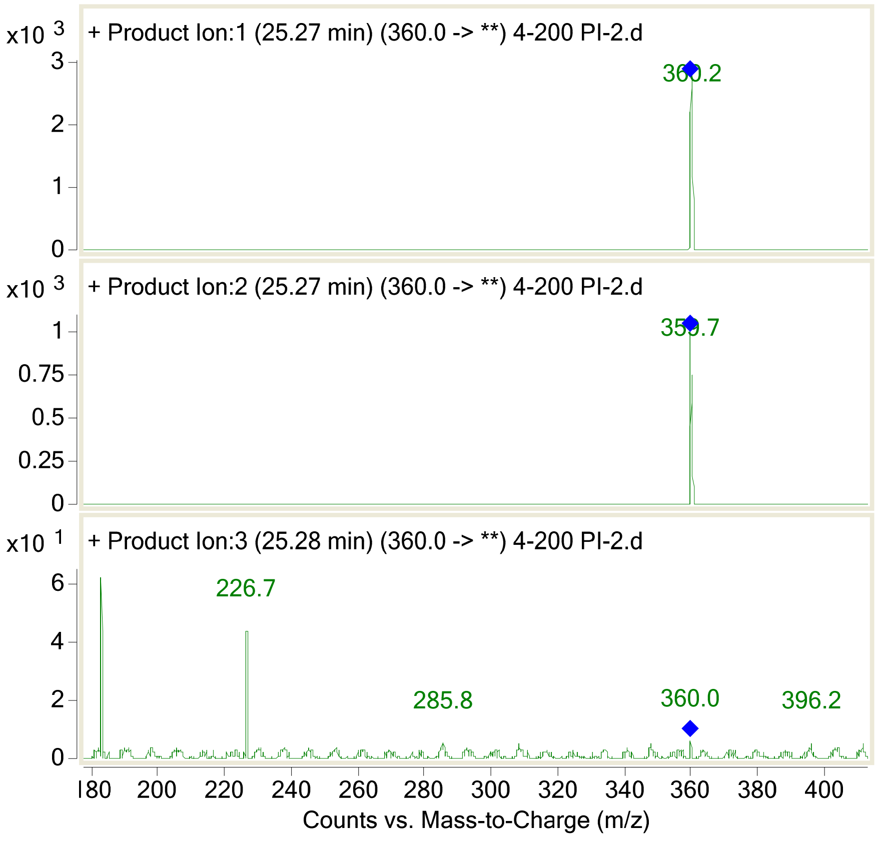

Spectral data of compounds **1**, **2**, **3**, **5**, **6**

Compound **1** (Calyciphyllin B), ESI-MS *m/z* 358.2 $[M+H]^+$. ^{1}H-NMR (300 MHz, CD_3OD) δ: 6.10 (1H, brs, H-15), 4.10 (1H, d, J = 6.4 Hz, H-1), 4.12 (1H, s, H-7), 3.15 (1H, m, H-10), 3.06 (1H, m, H-2), 2.60 (1H, m, H-14b), 2.58 (1H, m, H-6), 2.50 (1H, m, H-14a), 2.37 (2H, m, H-16), 1.13 (3H, d, J = 6.5 Hz, 20-Me), 1.35 (3H, s, 21-Me); ^{13}C-NMR (CD_3OD, 75 MHz) δ: 176.6 (C-22), 140.8 (C-9), 136.2 (C-15), 96.6 (C-1), 87.9 (C-5), 74.9 (C-19), 69.8 (C-7), 52.9 (C-8), 47.8 (C-2), 47.3 (C-6), 42.7 (C-10), 36.7 (C-4), 34.8 (C-18), 34.7 (C-17), 33.9 (C-16), 31.8 (C-13), 31.4 (C-11), 29.1 (C-14), 21.8 (C-3), 20.8 (C-12), 20.8 (C-21), 12.3 (C-20).

Compound **2** (Deoxycalyciphyllin B), m. p. 179.1–180.5 °C. ESI-MS *m/z* 342.2 $[M+H]^+$. ^{1}H-NMR (300 MHz, MeOD) δ: 5.48 (1H, d, J = 2.1 Hz, H-15), 3.82 (1H, d, J = 6.9 Hz, H-1), 3.11 (1H, d, J = 3.9 Hz, H-19b), 2.98 (1H, m, J = 7.5 Hz, H-10), 1.38 (3H, s, 21-Me), 1.02 (3H, d, J = 6.6 Hz, 20-Me). ^{13}C-NMR (75 MHz, MeOD) δ: 174.8 (C-22), 147.4 (C-9), 129.7 (C-15), 87.3 (C-5), 75.5 (C-1), 61.4 (C-19), 60.8 (C-7), 50.1 (C-8), 49.0 (C-6), 44.2 (C-2), 42.8 (C-10), 36.8 (C-4), 33.9 (C-18), 33.2 (C-17), 31.6 (C-16), 31.5 (C-13), 30.2 (C-11), 28.9 (C-14), 26.9 (C-3), 25.4 (C-12), 23.4 (C-21), 22.5 (C-3), 14.6(C-20).

Compound **3** (Daphnezomine M), ESI-MS *m/z* 346.2 $[M+H]^+$. ^{13}C-NMR(75 MHz, $CDCl_3$) δ: 177.5 (C-22), 59.8 (C-7), 54.0 (C-9), 52.5 (C-1), 50.5 (C-10), 46.8 (C-6), 42.9 (C-2), 41.5 (C-11), 39.3 (C-4),

38.5 (C-8), 38.2 (C-5), 37.1 (C-17), 31.7 (C-14), 30.9 (C-15), 29.8 (C-18), 28.9 (C-13), 27.6 (C-16), 24.9 (C-12), 22.0 (C-21), 21.7 (C-20), 21.5 (C-19), 21.2 (C-3).

Compound **5** (Methyl homosecodaphniphyllate), ESI-MS *m/z* 360.2 $[M+H]^+$. ^{13}C-NMR(75 MHz, $CDCl_3$) δ: 176.9 (C-22), 60.0 (C-7), 55.1 (C-9), 53.8 (C-1), 52.8 (C-23), 49.9 (C-10), 48.7 (C-6), 43.5 (C-2), 42.0 (C-11), 41.0 (C-4), 40.4 (C-17), 37.8 (C-14), 37.7 (C-8), 37.2 (C-5), 35.4 (C-15), 29.3 (C-18), 27.8 (C-13), 26.2 (C-16), 22.9 (C-12), 21.8 (C-20), 20.9 (C-19), 20.6 (C-21), 20.2 (C-3).

Compound **6 (**Daphiodhanins D), m. p. 110.6–112.1 °C. ESI-MS *m/z* 514.5 $[M+H]^+$. ^{1}H-NMR (300 MHz, DMSO-d_6) δ: 5.95 (1H, d, J = 6.6 Hz, H-25), 4.62 (1H, m, H-26), 3.05 (1H, s, H-1), 2.44 (1H, d, J = 4.8 Hz, H-7), 1.99 (3H, s, -OAc), 1.40 (3H, s, 30-Me), 0.90 (3H, d, J = 6.6 Hz, 19-Me), 0.86 (3H, d, J = 6.6 Hz, 20-Me), 0.85 (3H, s, 24-Me), 0.73 (3H, s, 21-Me). ^{13}C-NMR (75 MHz, DMSO-d_6) δ: 169.7 (-OAc), 98.3 (C-25), 82.4 (C-29), 73.2 (C-26), 59.5 (C-7), 54.3 (C-9), 51.0 (C-22), 50.3 (C-10), 49.5 (C-23), 47.3 (C-1), 46.7 (C-6), 43.2 (C-2), 39.0 (C-17), 38.8 (C-4), 36.4 (C-8), 36.0 (C-11), 35.5 (C-5), 33.6 (C-14), 28.3 (C-28), 28.0 (C-18), 27.6 (C-16), 25.5 (C-27), 23.5 (C-30), 22.7 (C-13), 21.2 (-OAc), 21.1 (C-19), 21.0 (C-20), 20.5 (C-12), 20.1 (C-3), 20.0 (C-21), 16.9 (C-24).
